# Docosahexaenoic Acid Alleviates Palmitic Acid-Induced Inflammation of Macrophages via TLR22-MAPK-PPARγ/Nrf2 Pathway in Large Yellow Croaker (*Larimichthys crocea*)

**DOI:** 10.3390/antiox11040682

**Published:** 2022-03-31

**Authors:** Dan Xu, Kun Cui, Qingfei Li, Si Zhu, Junzhi Zhang, Shengnan Gao, Tingting Hao, Kangsen Mai, Qinghui Ai

**Affiliations:** 1 Key Laboratory of Aquaculture Nutrition and Feed (Ministry of Agriculture and Rural Affairs), Key Laboratory of Mariculture (Ministry of Education), Ocean University of China, 5 Yushan Road, Qingdao 266003, China; danxu_ouc@163.com (D.X.); 15764210558@163.com (K.C.); qfli0310@163.com (Q.L.); dscdove@163.com (S.Z.); zhjun123@hunau.edu.cn (J.Z.); gaoshengnan21@gmail.com (S.G.); rachelhaotingting@163.com (T.H.); kmai@ouc.edu.cn (K.M.); 2 Laboratory for Marine Fisheries Science and Food Production Processes, Qingdao National Laboratory for Marine Science and Technology, 1 Wenhai Road, Qingdao 266237, China

**Keywords:** palmitic acid, inflammatory response, Toll-like receptor, docosahexaenoic acid, *Larimichthys crocea*

## Abstract

Palmitic acid (PA) is a saturated fatty acid (SFA) that can cause an inflammatory response, while docosahexaenoic acid (DHA) is always used as a nutritional modulator due to its anti-inflammatory properties. However, the potential molecular mechanism is still not completely elucidated in fish. Herein, the PA treatment induced an inflammatory response in macrophages of large yellow croaker (*Larimichthys crocea*). Meanwhile, the mRNA expression of Toll-like receptor (TLR)-related genes, especially *tlr22*, and the phosphorylation of the mitogen-activated protein kinase (MAPK) pathway were significantly upregulated by PA. Further investigation found that the PA-induced inflammatory response was suppressed by *tlr22* knockdown and MAPK inhibitors. Moreover, the results of the peroxisome proliferator-activated receptor γ (PPARγ) agonist and inhibitor treatment proved that PPARγ was involved in the PA-induced inflammation. PA treatment decreased the protein expression of PPARγ, while *tlr22* knockdown and MAPK inhibitors recovered the decreased expression. Besides, the PA-induced activation of Nrf2 was regulated by p38 MAPK. Furthermore, DHA-executed anti-inflammatory effects by regulating the phosphorylation of the MAPK pathway and expressions of PPARγ and Nrf2. Overall, the present study revealed that DHA alleviated PA-induced inflammation in macrophages via the TLR22-MAPK-PPARγ/Nrf2 pathway. These results could advance the understanding of the molecular mechanism of the SFA-induced inflammatory response and provide nutritional mitigative strategies.

## 1. Introduction

Palmitic acid (PA), one of the most common saturated fatty acids (SFAs), can be transported in cells and converted into phospholipids, diacylglycerol and ceramides [1,2,3]. PA has been reported to induce an inflammatory response of macrophages in mammals, which triggers the activation of various signaling pathways and induces the production of cytokines [4]. Palm oil (PO), enriched with PA, is increasingly used as an alternative to fish oil in aquaculture [5]. Although partially replacing fish oil with PO in diets has no significant effects on the growth performance of cultured fish, the overuse of PO often induces an inflammatory response and suppresses the antioxidant capacity [6,7]. Moreover, PA has been proven to induce an inflammatory response in fish [8]. However, the molecular mechanism of the inflammatory response induced by PA is still not completely understood.

As a family of pattern recognition receptors (PRRs), Toll-like receptors (TLRs) play an essential role in initiating and regulating innate immunity both in mammals and fish [9,10]. The activation of TLRs recruits its adaptor proteins, such as the myeloid differentiation factor 88 (MyD88) and TIR domain containing adaptor-inducing interferon-β (TRIF). Then, downstream signaling cascades are triggered and activated, which could induce the expression of inflammatory genes [11,12]. Mitogen-activated protein kinase (MAPK) and nuclear transcription factor kappa-B (NF-κβ) are the widely studied downstream pathways of TLR. In mammals, PA could act as a strong agonist of TLR2 and TLR4 and activate downstream inflammatory signaling pathways [13,14]. However, the composition of the TLR family in teleost is different from that in mammals, which has some members considered to be fish-specific TLRs. Among all the fish-specific TLRs, TLR22 is widely explored as the typical member in many fish species [15,16,17,18]. Previous studies on large yellow croaker (*Larimichthys crocea*) have revealed that TLR22 could respond to fatty acids [19,20]. However, the role and downstream regulation mechanism of TLR22 in the PA-induced inflammatory response remain unclear in fish.

N-3 polyunsaturated fatty acids (PUFAs), such as docosahexaenoic acid (DHA) and eicosapentaenoic acid (EPA), can inhibit inflammation by regulating the activity of inflammatory signaling pathways and influencing the production of lipid mediators [21,22]. PUFAs could inhibit SFA-induced cyclooxygenase-2 expression by mediating a common signaling pathway derived from TLR [23]. Moreover, PUFA supplementation in diets results in a lower incidence of metabolic disease [24]. Previous studies on large yellow croaker have revealed that DHA supplementation in diets significantly improved fish health, and the anti-inflammatory effect of DHA was much stronger than EPA [25,26]. However, the anti-inflammatory molecular mechanism of DHA in fish needs further investigation.

Large yellow croaker is an important mariculture fish in China. In large yellow croaker feed, PO is widely used as a promising alternative to fish oil. However, it is still limited in the mechanism of inflammatory response induced by PA. Nutritional regulation of the immune system provides a potentially powerful strategy for improving fish health and quality. Studies on nutritional regulation in large yellow croaker have been intensively explored [27,28]. Large yellow croaker can be used as a good model animal in nutrition research. Therefore, this study aimed to investigate the molecular mechanism of PA-induced inflammation and its mitigative strategy mediated by DHA in macrophages of large yellow croaker. These results could advance the understanding of the molecular mechanism of the inflammatory response induced by SFAs and provide nutritional strategies against inflammation.

## 2. Materials and Methods

### 2.1. Macrophages Culture and Treatment

Large yellow croaker (weight 500 ± 50.26 g) was purchased from a commercial fish farm in Ningbo, China. All experimental procedures performed on fish were in strict accordance with the Management Rule of Laboratory Animals (Chinese Order No. 676 of the State Council, revised 1 March 2017). Macrophages were isolated from fish head kidneys and maintained according to the previous procedure with some modifications [29]. The primary macrophage was cultured in DMEM/F12 medium supplemented with 10% fetal bovine serum (FBS; Gibco, Carlsbad, CA, USA), 100-U/mL penicillin and 100-mg/mL streptomycin at 28 °C. Before stimulation, macrophages were seeded in six-well plates (Corning, Corning, NY, USA) at 2.0 × 10^6^ cells per well. PA (Sigma-Aldrich, St. Louis, MO, USA) was combined with 1% fatty acid-free bovine serum albumin (BSA; Equitech-Bio, Kerrville, TX, USA) to reach a final concentration at 1 mm. Before the fatty acids treatment, cells were starved with DMEM/F12 alone for 1 h. Macrophages were incubated with different concentration of PA at 250 or 500 µM for 12 h. Cells treated with 1% fatty acid-free BSA were considered as the control group.

To confirm the role of the MAPK pathway in PA-induced inflammation, SB203580 (p38 inhibitor; MCE, Jersey City, NJ, USA) and SP600125 (JNK inhibitor; MCE) were used at a final concentration of 10 μm for 2 h before PA treatment. Furthermore, to confirm the role of PPARγ in PA-induced inflammation, troglitazone (PPARγ agonist; MCE) and GW9662 (PPARγ inhibitor; MCE) were incubated at a final concentration of 10 μm for 12 h before PA treatment. The control group was incubated with the same concentration of dimethyl sulfoxide (DMSO; Solarbio, Beijing, China).

To investigate the effect of DHA (Sigma-Aldrich, St. Louis, MO, USA) on PA-induced inflammation, macrophages were incubated with 200-μm DHA for 12 h before PA stimulation. After culturing, the macrophages were harvested for further analysis.

### 2.2. RNA Isolation and Quantitative Real-Time PCR (RT-qPCR)

Total RNA was extracted from macrophages by RNAiso Plus (Takara, Tokyo, Japan) according to the manufacturer’s instructions. The 1.5% denaturing agarose gel was used to measure the integrity of the RNA. The concentration and quality of extracted total RNA were confirmed by a NanoDrop^®^2000 spectrophotometer (Thermo Scientific, Waltham, MA, USA). Then, complementary DNA (cDNA) was reverse-transcribed from the extracted RNA with the PrimeScript™ RT reagent kit (Takara, Tokyo, Japan).

The RT-qPCR primers used in this study are shown in Table 1 according to the nucleotide sequences of target genes in large yellow croaker. *β-actin* was used as the housekeeping gene. RT-qPCR was performed on a CFX96 Touch real-time PCR detection system (Bio-Rad, Hercules, CA, USA) using the SYBR Premix Ex Taq kit (Takara, Tokyo, Japan). The amplification was performed in a total volume of 20 μL, containing 10 μL of SYBR qPCR Master Mix, 6 μL of DEPC water, 2 μL of cDNA and 1 μL of F/R primer. The PCR temperature profile was performed at 95 °C for 2 min and, afterward, 39 cycles of 95 °C for 10 s, 58 °C for 15 s and 72 °C for 10 s. The gene expression levels were calculated via the 2^−ΔΔCT^ method [30].

### 2.3. Flow Cytometry Analysis

Macrophages treated with PA and BSA (the control group) were harvested and incubated with CD68 or CD209 antibodies at 37 °C for 1 h. CD68 and CD209 antibodies were produced by immunizing rabbits with synthetic recombinant proteins according to sequences from large yellow croaker, and the specificity was verified in previous studies [31]. Alexa Flour 488 goat anti-rabbit IgG (Beyotime Biotechnology, Shanghai, China) was incubated for 45 min at 37 °C to combine with primary antibodies. The percentage of positive cells was detected, and the data was analyzed by a flow cytometer (BD Accuri^TM^ C6, Franklin Lakes, NJ, USA). Gated represent macrophages (R1) were selected. M1 and M2 represented CD68^+^ and CD209^+^ populations compared with the control group, respectively.

### 2.4. Western Blotting

RIPA reagent (Solarbio, Beijing, China) with a supplementation of protease and phosphatase inhibitors (Thermo Fisher Scientific, USA) was used to obtain the proteins of macrophages. Protein concentrations were measured by the BCA Protein Assay Kit (Beyotime Biotechnology, Shanghai, China). All protein concentrations were adjusted to the same level before heating. Western blot experiments were performed as follows: 20 μg of proteins were loaded on 10% SDS-PAGE and then transferred to polyvinylidene difluoride (PVDF) membranes (Merck Millipore, Berlin, Germany). The PVDF membranes were incubated with 5% skim milk for 2 h at room temperature and then incubated with the targeting antibody overnight at 4 °C. The primary antibodies against ERK1/2 (Cat. No. 4695), phospho-ERK1/2 (Cat. No. 4370), JNK1/2 (Cat. No. 9252), phospho-JNK1/2 (Cat. No. 4668), p38 (Cat. No. 8690), phospho-p38 (Cat. No. 9215), IKKβ (Cat. No. 2678), phospho-IKKα/β (Cat. No. 2697) and PPARγ (Cat. No. 2443) were obtained from Cell Signaling Technology (Boston, MA, USA). Anti-GAPDH antibody (Cat. No. TA-08; Golden Bridge Biotechnology, Beijing, China) was used as the reference. Then, the membrane was incubated with the secondary antibody (HRP-labeled Goat Anti-Rabbit IgG (H + L)) for 2 h at room temperature and then was visualized by an electrochemiluminescence kit (Beyotime Biotechnology, Shanghai, China). The target proteins were quantified using ImageJ software (National Institutes of Health, Bethesda, MD, USA).

### 2.5. RNA Interference

Large yellow croaker TLR22-specific small interfering RNA (siRNA; sense and antisense sequences of siRNA are shown in Table 1; Gene Pharma, Shanghai, China) was transfected into cells using the Xfect™ RNA Transfection Reagent (Takara, Tokyo, Japan) according to the manufacture’s protocol for 36 h to knock down the expression of *tlr22* in macrophages. The RNAi negative control (NC) was used as the control group. After transfection for 36 h, cells were treated with PA for another 12 h.

### 2.6. Statistical Analysis

SPSS 22.0 (IBM, Armonk, NY, USA) was used to perform the statistical analysis, and the results were presented as means ± standard error of the mean (S.E.M.). All data were subjected to independent sample *t*-tests or one-way analysis of variance (ANOVA) followed by Tukey’s multiple range test. A value of *p* < 0.05 was considered statistically significant.

## 3. Results

### 3.1. PA-Induced Inflammatory Response in Macrophages of Large Yellow Croaker

**Figure 1:**
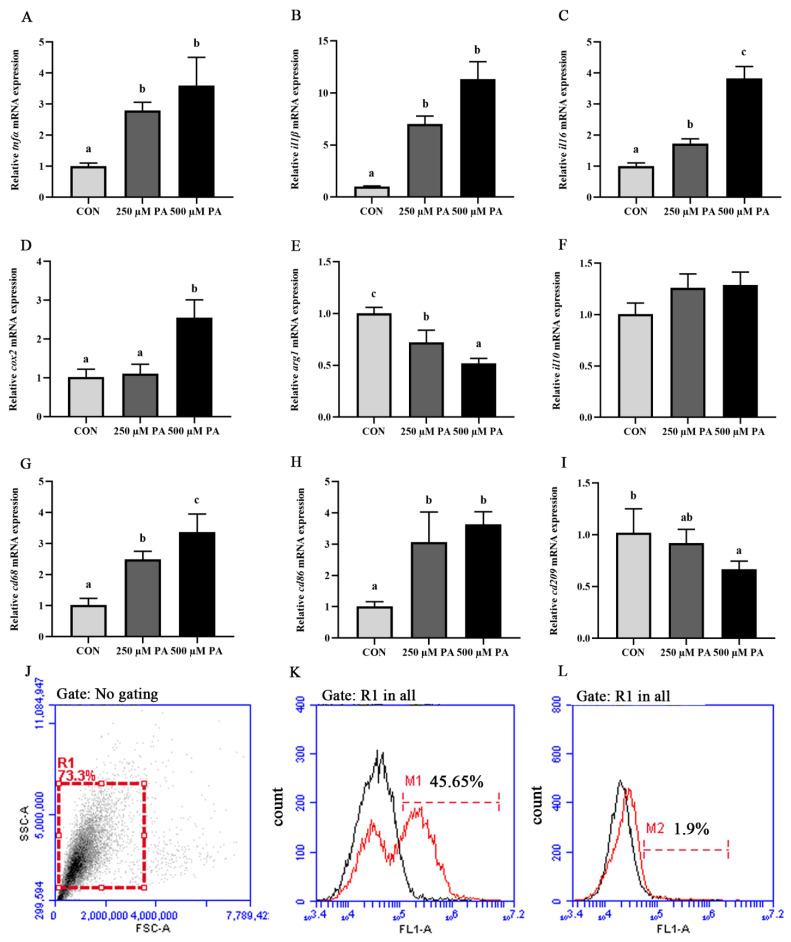
PA induced inflammatory response in macrophages of large yellow croaker. (**A**–**I**) mRNA expression levels of inflammatory genes (*tnfα*, *il1β*, *il6*, *cox2*, *arg1* and *il10*) and macrophage markers (*cd68*, *cd86* and *cd209*) after PA treatment (*n* = 6). (**J**–**L**) Fluorescence histograms of CD68^+^ (**K**) and CD209^+^ (**L**) populations after PA treatment (scale of M1). Represent macrophages gated (R1) on a forward scatter (FSC) versus side scatter (SSC) dot plot. Data are presented as the means ± SEM and are analyzed using one-way ANOVA, followed by Tukey’s test. Bars labeled with the same letters are not significantly different (*p* > 0.05).

The PA treatment significantly upregulated the mRNA expression levels of proinflammatory genes, including *tnfα*, *il1β*, *il6* and *cox2* (*p* < 0.05) (Figure 1A–D). The mRNA expression level of anti-inflammatory gene *arg1* was significantly decreased in the PA treatment compared with that in the control group (*p* < 0.05), while the mRNA expression of *il10* showed no significant differences (*p* > 0.05) (Figure 1E,F). Moreover, mRNA expression levels of *cd68* and *cd86*, markers of proinflammatory phenotype macrophages, were significantly increased after PA treatment, whereas the mRNA expression of *cd209*, a marker of the anti-inflammatory phenotype macrophage, was significantly decreased (*p* < 0.05) (Figure 1G–I). Gated macrophages (R1) in forward and side scatter (FS-SS) dot plots and combined fluorescence histograms were shown (Figure 1J). The flow cytometry analysis showed that the PA treatment significantly increased the CD68^+^ population (Figure 1K,L). These results demonstrated that PA treatment induced an inflammatory response in macrophages of large yellow croaker.

### 3.2. PA Activated the TLR-Related Genes Expression and MAPK Signaling Pathway in Macrophages

**Figure 2:**
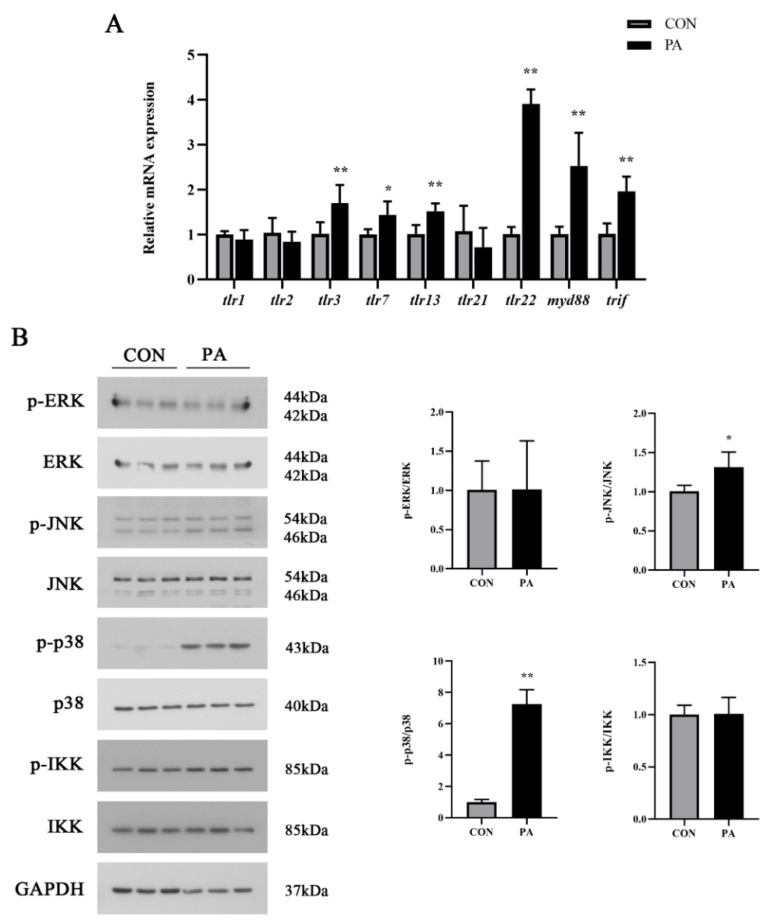
PA activated the TLR-related genes expression and MAPK signaling pathway in macrophages. (**A**) The mRNA expression level of TLR-related genes after PA treatment. (**B**) Western blot analysis of MAPK signaling activation after PA treatment. The ratios of p-ERK to ERK, p-p38 to p38, p-JNK to JNK and p-IKK to IKK were determined. The data are presented as the means ± SEM (*n* = 6) and are analyzed using independent *t*-tests. * *p* < 0.05 and ** *p* < 0.01 indicate significant differences compared with the control group.

To investigate whether the TLR signaling pathway was activated by PA, TLR-related genes expression and downstream signaling pathways were detected in macrophages after PA treatment. PA treatment significantly upregulated the mRNA expression levels of *tlr3*, *tlr7*, *tlr13*, *tlr22*, *myd88* and *trif* (*p* < 0.05), while the mRNA expression levels of *tlr1*, *tlr2* and *tlr21* had no significant changes (*p* > 0.05) (Figure 2A). As a typical specific TLR, the increased expression level of *tlr22* was more conspicuous than the others. Moreover, the phosphorylation level of MAPK, including JNK and p38, was significantly increased after the PA treatment (*p* < 0.05), while the phosphorylation levels of ERK and IKK showed no significant differences (*p* > 0.05) (Figure 2B). These results revealed that the PA treatment induced TLRs gene expressions, especially *tlr22*, and activated the MAPK pathway in macrophages.

### 3.3. Effects of TLR22-MAPK Signaling Pathway on PA-Induced Inflammation in Macrophages

**Figure 3:**
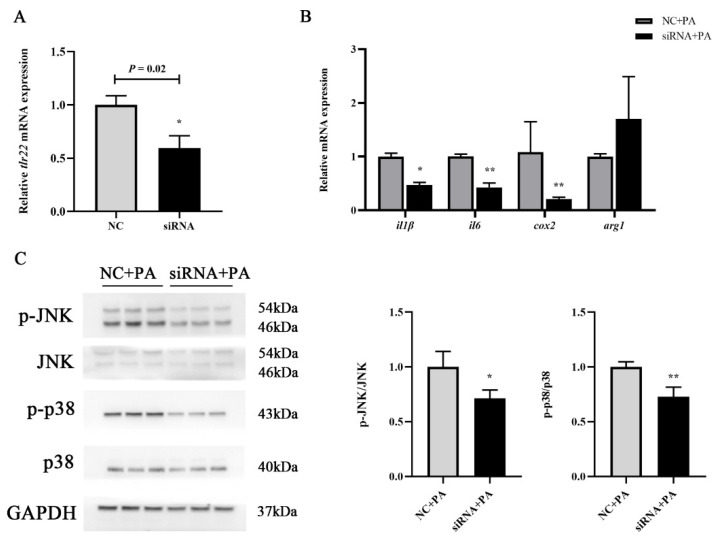
Effects of *tlr22* knockdown on PA-induced inflammation in macrophages. (**A**) The mRNA expression of *tlr22* after *tlr22* knockdown. (**B**) Effects of *tlr22* knockdown on the mRNA expression levels of inflammatory genes induced by PA. (**C**) Effects of *tlr22* knockdown on the phosphorylation of the MAPK pathway induced by PA. The ratios of p-p38 to p38 and p-JNK to JNK were determined. The data are presented as the means ± SEM (*n* = 3) and are analyzed using independent *t*-tests. * *p* < 0.05 and ** *p* < 0.01 indicate significant differences compared with the control group.

**Figure 4:**
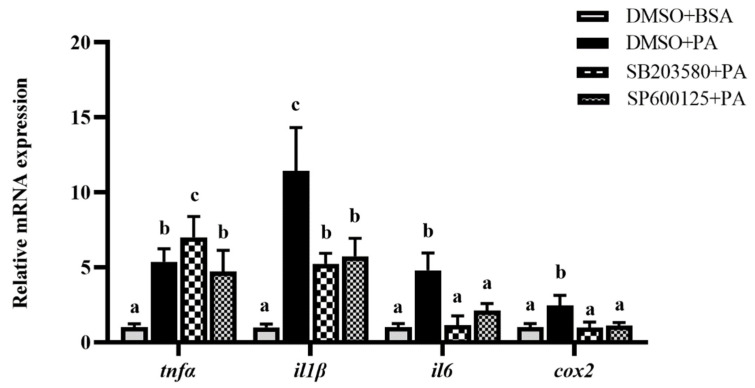
Effects of p38 (SB203580) and JNK (SP600125) inhibitors on the mRNA expression levels of inflammatory genes induced by PA. Data are presented as the means ± SEM (*n* = 6) and are analyzed using Tukey’s test. Bars labeled with the same letters are not significantly different (*p* > 0.05).

To confirm whether the TLR22-MAPK pathway participated in PA-induced inflammation, TLR22 siRNA was transfected, and MAPK inhibitors were incubated in macrophages. The transfection of siRNA into macrophages significantly inhibited the *tlr22* mRNA expression (*p* < 0.05) (Figure 3A). Meanwhile, *tlr22* knockdown significantly suppressed the PA-induced mRNA expression levels of proinflammatory genes, including *il1β*, *il6* and *cox2* (*p* < 0.05), while the mRNA expression level of *arg1* showed no significant differences (*p* > 0.05) (Figure 3B). As the downstream pathway of TLR, the change of MAPK was detected after *tlr22* knockdown under PA treatment. The results showed that the PA-induced phosphorylation levels of JNK and p38 were significantly inhibited after *tlr22* knockdown (*p* < 0.05) (Figure 3C). Furthermore, JNK and p38 inhibitor incubation significantly suppressed the PA-induced mRNA expression levels of proinflammatory genes, including *il1β*, *il6* and *cox2* (*p* < 0.05) (Figure 4). These results demonstrated that PA induced an inflammatory response of macrophages via the TLR22-MAPK signaling pathway.

### 3.4. PPARγ Participated in PA-Induced Inflammation via TLR22-MAPK Pathway

**Figure 5:**
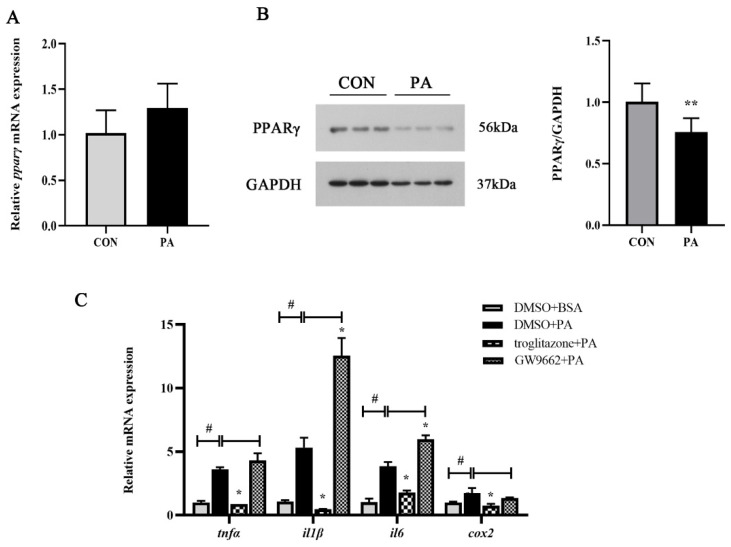
PPARγ was involved in PA-induced inflammation. (**A**,**B**) mRNA and protein expression levels of PPARγ after PA treatment (*n* = 6). The ratio of PPARγ to GAPDH was determined. (**C**) Effects of the PPARγ activator (troglitazone) and inhibitor (GW9662) on the mRNA expression levels of the inflammatory genes induced by PA (*n* = 3). Data are presented as the means ± SEM and analyzed using independent *t*-tests. # *p* < 0.05 indicates significant differences compared with the BSA group as the negative control. * *p* < 0.05 and ** *p* < 0.01 indicate significant differences compared with the control group.

**Figure 6:**
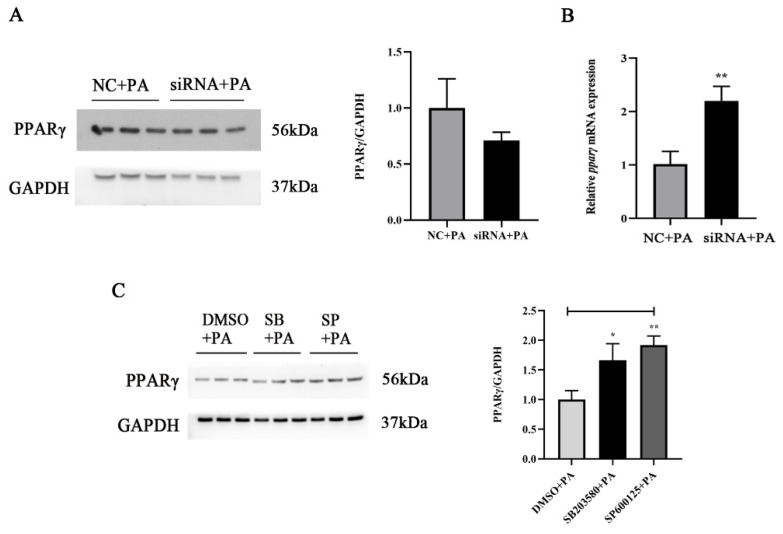
PPARγ participated in PA-induced inflammation via the TLR22-MAPK pathway. (**A**,**B**) Effects of *tlr22* knockdown on the protein and mRNA expression levels of PPARγ induced by PA (*n* = 3). (**C**) Effects of the p38 (SB203580) and JNK (SP600125) inhibitors on the protein expression level of PPARγ induced by PA (*n* = 3). Data are presented as the means ± SEM and analyzed using independent *t*-tests. * *p* < 0.05 and ** *p* < 0.01 indicate significant differences compared with the control group.

As an integral part of inflammatory responses, we further investigated whether PPARγ was involved in regulating PA-induced inflammation. The PA treatment significantly decreased the protein expression level of PPARγ (*p* < 0.05), while the mRNA expression level of *pparγ* showed no significant differences (*p* > 0.05) (Figure 5A,B). The PPARγ agonist and inhibitor were used to explore the role of PPARγ in PA-induced inflammation. The activation of PPARγ significantly suppressed the PA-induced mRNA expression levels of proinflammatory genes, including *tnfα*, *il1β*, *il6* and *cox2* (*p* < 0.05) (Figure 5C). Meanwhile, the inhibition of PPARγ significantly enhanced the mRNA expression levels of *il1β* and *il6* (*p* < 0.05) (Figure 5C). Moreover, *tlr22* knockdown significantly enhanced the mRNA expression level of *pparγ* induced by PA (*p* < 0.05) but was not significantly different in the protein expression (*p* > 0.05) (Figure 6A,B). However, p38 and JNK inhibitor incubation significantly enhanced the protein expression level of PPARγ induced by PA (*p* < 0.05) (Figure 6C). These results revealed that PPARγ participated in PA-induced inflammation via the TLR22-MAPK pathway.

### 3.5. p38 MAPK Regulated the PA-Induced Activation of Nrf2

**Figure 7:**
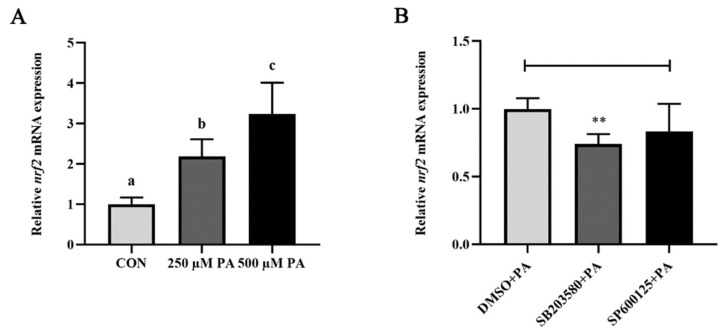
p38 MAPK regulated the PA-induced activation of Nrf2. (**A**) The mRNA expression level of *nrf2* after PA treatment. (**B**) Effects of the p38 (SB203580) and JNK (SP600125) inhibitors on the mRNA expression level of *nrf2* induced by PA. Data are presented as the means ± SEM (*n* = 6) and analyzed using one-way ANOVA, followed by Tukey’s test and independent *t*-tests. Bars labeled with the same letters are not significantly different (*p* > 0.05 and ** *p* < 0.01).

Nrf2, which was widely known to be the major regulation of antioxidant processes, also played a role in regulating inflammation. The PA treatment significantly upregulated the mRNA expression level of *nrf2* (*p* < 0.05) (Figure 7A). p38 inhibitor incubation significantly suppressed the PA-induced mRNA expression level of *nrf2* (*p* < 0.05), while the JNK inhibitor had no effects (Figure 7B). These results showed that p38 MAPK regulated the PA-induced activation of Nrf2.

### 3.6. The Protective Effect of DHA against PA-Induced Inflammation

**Figure 8:**
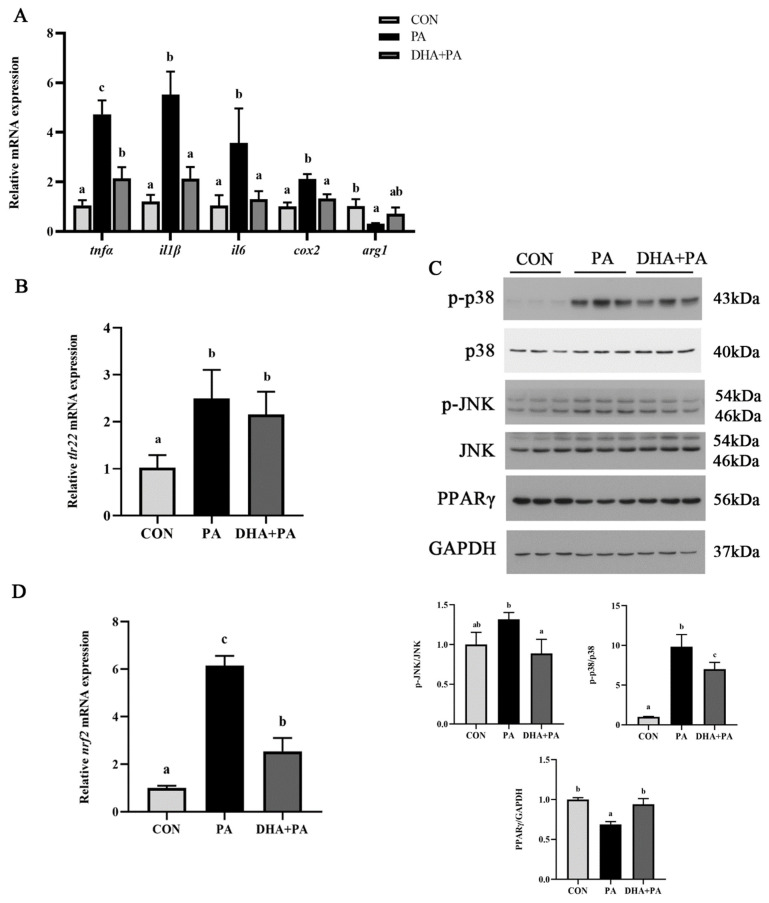
The protective effect of DHA against PA-induced inflammation. (**A**) mRNA expression levels of inflammatory genes after DHA treatment (*n* = 4). (**B**) The mRNA expression level of *tlr22* after DHA treatment (*n* = 4). (**C**) Western blot analysis of MAPK signaling activation and the PPARγ protein level after DHA treatment (*n* = 3). The ratios of p-p38 to p38, p-JNK to JNK and PPARγ to GAPDH were determined. (**D**) The mRNA expression level of *nrf2* after DHA treatment (*n* = 4). Data are presented as the means ± SEM and analyzed using one-way ANOVA, followed by Tukey’s test. Bars labeled with the same letters are not significantly different (*p* > 0.05).

N-3 PUFAs are widely known to exert anti-inflammatory effects. Hence, the protective effect of DHA against PA-induced inflammation in macrophages was investigated. DHA treatment significantly suppressed the PA-induced mRNA expression levels of proinflammatory genes, including *tnfα*, *il1β*, *il6* and *cox2* (*p* < 0.05), while the mRNA expression of anti-inflammatory gene *arg1* was significantly upregulated (*p* < 0.05) (Figure 8A). However, the mRNA expression level of *tlr22* had no significant differences after DHA treatment (*p* > 0.05) (Figure 8B). Furthermore, the DHA treatment significantly reduced the PA-induced phosphorylation levels of JNK and p38 (*p* < 0.05) (Figure 8C). Moreover, DHA significantly recovered the decrease of PPARγ protein expression caused by PA (*p* < 0.05) (Figure 8C). DHA also significantly inhibited the increase of *nrf2* mRNA expression induced by PA (*p* < 0.05) (Figure 8D). These results demonstrated that the protective effect of DHA against the PA-induced inflammatory response could perform via the TLR22-MAPK-PPARγ/Nrf2 signaling pathway.

## 4. Discussion

This study utilized the primary head kidney macrophages, an important immune cell in fish, to investigate the potential molecular mechanism of the PA-induced inflammatory response. In the present study, we found that PA induced proinflammatory gene expressions and promoted proinflammatory macrophage polarization, which was consistent with the observations in mammals [32,33].

Fish rely more on their innate immune system to resist the invasion of bacteria because of their evolutionarily less well-developed adaptive immune system [34]. One of mechanisms by which the innate immune system senses stimulation is through TLR signaling. As the TLR family in teleost was different from that in mammals, the change of TLR-related genes in large yellow croaker was detected according to a previous study [19]. The results showed that PA significantly activated the expressions of TLRs and adaptor proteins. Among them, TLR22 may act as the main response receptor to PA. However, TLR22 orthologs in humans serve as nonfunctional pseudogenes [35]. Furthermore, TLR22 knockdown suppressed PA-induced inflammation in macrophages. The current study, for the first time, revealed the role of TLR22 in PA-induced inflammation in fish. Further investigation found that PA induced the activation of JNK and p38 pathways. Although, a previous study in large yellow croaker showed that PO and PA could activate the NF-κβ pathway in the liver and hepatocytes, the present study found that PA activated MAPK but not NF-κβ in macrophages [36,37]. Meanwhile, the transcriptome analysis in the spleen of large yellow croaker revealed multiple signaling pathways were involved in the antiviral response, including the MAPK, NF-β and JAK pathways [38,39]. Hence, previous studies do not have uniform results on the changes of downstream signaling in response to different stimulations [40,41,42]. These inconsistent results may be due to the fact that different tissues had different regulation mechanisms of TLRs in response to different stimulations. Therefore, the signaling pathways regulated by TLR22 in different stimulations remain to be further explored in fish.

Since PPARγ could be modulated by TLRs and perform an anti-inflammatory effect, we further confirmed whether PPARγ was involved in PA-induced inflammation. The results showed that PA decreased the PPARγ expression at the translation level. The effects of the agonist and inhibitor of PPARγ on inflammatory gene expressions indicated that it presented an anti-inflammatory effect in regulating PA-induced inflammation, which was consistent with previous studies in mammals [43]. Previous studies in large yellow croaker revealed that PPARγ could negatively regulate the expression of inflammatory genes by affecting the promoter activity of genes [44]. Furthermore, the *tlr22* knockdown and MAPK inhibitors recovered the decrease of PPARγ expression caused by PA. These results demonstrated that PPARγ participated in PA-induced inflammation via the TLR22-MAPK pathway in macrophages of large yellow croaker.

There is a complex interaction between the inflammatory response and antioxidant systems. Nrf2 is an important transcriptional regulator regulating the expression of varieties of anti-inflammatory and antioxidant genes and plays a central role in the response to stimulation [45]. In the current study, Nrf2 could be activated by PA, and p38 regulated the PA-induced activation of Nrf2. The results in mammals have reported that Nrf2 could participate in the alleviation of chronic inflammation by the MAPK pathway [46,47]. Therefore, the PA-induced activation of Nrf2 in the present study may be to compete and suppress the inflammatory response induced by PA.

N-3 PUFAs perform anti-inflammatory and antioxidant properties through various mechanisms and are always used as nutritional regulation strategies in mammals [48,49]. After understanding the molecular mechanism of PA in regulating inflammation, we further investigated whether DHA could alleviate PA-induced inflammation. The results showed that DHA inhibited the effect of PA on the mRNA expression of proinflammatory genes and the phosphorylation of JNK and p38, which was consistent with the results in mammals [50,51]. However, the mRNA expression of *tlr22* showed no differences. Previous studies in mammals suggested that the effect of PUFAs on impacting TLR activation might perform by regulating the lipid and protein compositions of the raft membrane, not directly affecting the TLR mRNA expression [52,53]. The results in our lab also found that DHA might regulate the activation of TLR22 by changing the biophysical properties of the cell membrane (unpublished data). Furthermore, DHA recovered the decrease of PPARγ protein expression induced by PA. DHA may affect PPARγ expression by regulating the signaling pathways or directly activating PPARγ as a ligand [54]. Together, these results revealed that the protective effect of DHA against PA-induced inflammation was executed through the TLR22-MAPK-PPARγ/Nrf2 signaling pathway.

## 5. Conclusions

**Figure 9:**
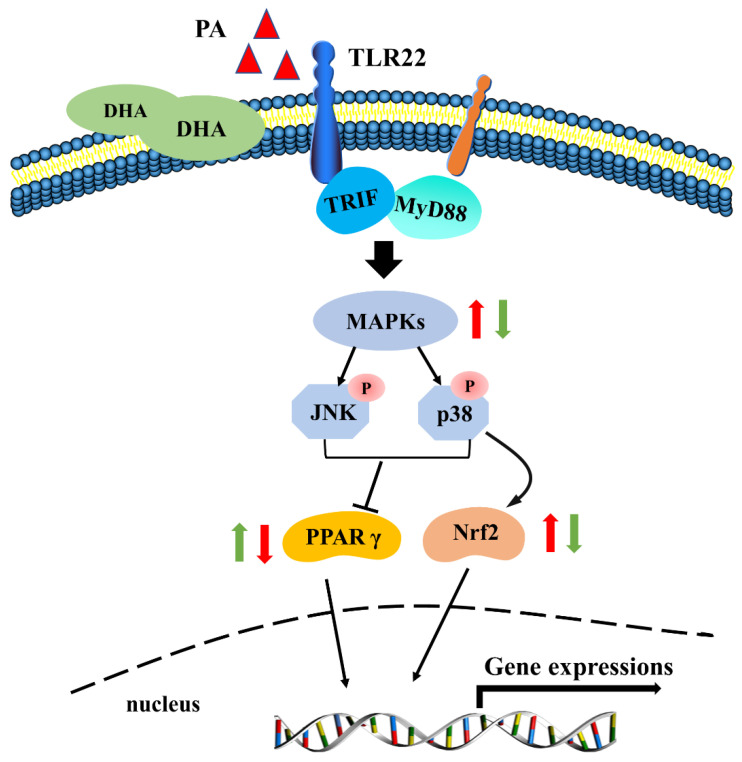
A working model showed the molecular mechanism of the PA-induced inflammatory response and the protective effect of DHA against the inflammation in macrophages of large yellow croaker.

In conclusion, the present study indicated the PA-induced inflammatory response of macrophages via the TLR22-MAPK-PPARγ/Nrf2 signaling pathway in large yellow croaker. Meanwhile, DHA was found to alleviate the PA-induced inflammation, thereby performing anti-inflammatory and antioxidation properties (Figure 9). These results could advance the understanding of the molecular mechanism of the inflammatory response induced by SFAs and provide nutritional strategies against inflammation, thereby improving the utilization rate of PO in aquafeed.

## Figures and Tables

**Table 1:** Primers used for RT-qPCR and the gene accession number.

Gene	Forward (5′-3′)	Reverse (5′-3′)	Accession Number
*β-actin*	GACCTGACAGACTACCTCATG	AGTTGAAGGTGGTCTCGTGGA	GU584189
*tnf* *α*	ACACCTCTCAGCCACAGGAT	CCGTGTCCCACTCCATAGTT	NM_001303385
*il1β*	CATAGGGATGGGGACAACGA	AGGGGACGGACACAAGGGTA	XM_010736551
*il6*	CGACACACCCACTATTTACAAC	TCCCATTTTCTGAACTGCCTC	XM_010734753
*cox2*	CTGGAAAGGCAACACAAGC	CGGTGAGAGTCAGGGACAT	XM_010734489
*arg1*	AACCACCCGCAGGATTACG	AAACTCACTGGCATCACCTCA	XM_19269015
*il10*	AGTCGGTTACTTTCTGTGGTG	TGTATGACGCAATATGGTCTG	XM_010738826
*cd68*	GCAGGGCTTCAATCTGACCAA	AGGATGAGCACCAGCAATGTC	NM_001319937
*cd86*	TGTGCGTCTTAGTCTACCTTCT	AAACTCTTCCGTCATCTTGC	XM_010756962
*cd209*	GATGGGTGTATTTCAGCGGTAG	TGTTGATAATCACCAGGTCTGC	XM_027278935
*tlr1*	TGTGCCACCGTTTGGATA	TTCAGGGCGAACTTGTCG	KF318376
*tlr2*	TCTGCTGGTGTCAGAGGTCA	GGTGAATCCGCCATAGGA	XM_027287556
*tlr3*	ACTTAGCCCGTTTGTGGAAG	CCAGGCTTAGTTCACGGAGG	XM_019274877
*tlr7*	ATGCAATGAGCCAAAGTCT	CATGTGAGTCAATCCCTCC	XM_010743042
*tlr13*	CCTCCTGTTTATGGTAGTGTCC	GCTCGTCATGGGTGTTGTAG	XM_010743101
*tlr21*	CTTTGCCTACATCACAGGGACT	GAAACACGAGCAGGAGAACATC	KY025428
*tlr22*	TATGCGAGCAGGAAGACC	CAGAAACACCAGGATCAGC	GU324977
*myd88*	TACGAAGCGACCAATAACCC	ATCAATCAAAGGCCGAAGAT	EU978950
*trif*	TACAATACTGTTATCCCTCTGCTGC	TCTCTTCTGTTTTCTAATCCTCGCG	MK863372
*pparγ*	TGTCCGAGCTGGAAGACAAC	TGGGGTCATAGGGCATACCA	XM_010731330
*nrf2*	GATGGAAATGGAGGTGATGC	CATGTTCTTTCTGTCGGTGG	XM_010737768
si*tlr22*	GCAAGUUUGGUGGUGCUUUTT	AAAGCACCACCAAACUUGCTT	GU324977

## Data Availability

Data is contained within the article.
